# Ebola Virus Infections in Nonhuman Primates Are Temporally Influenced by Glycoprotein Poly-U Editing Site Populations in the Exposure Material

**DOI:** 10.3390/v7122969

**Published:** 2015-12-19

**Authors:** John C. Trefry, Suzanne E. Wollen, Farooq Nasar, Joshua D. Shamblin, Steven J. Kern, Jeremy J. Bearss, Michelle A. Jefferson, Taylor B. Chance, Jeffery R. Kugelman, Jason T. Ladner, Anna N. Honko, Dean J. Kobs, Morgan Q.S. Wending, Carol L. Sabourin, William D. Pratt, Gustavo F. Palacios, M. Louise M. Pitt

**Affiliations:** 1 Virology Division, US Army Medical Research Institute for Infectious Diseases, 1425 Porter St., Fort Detrick, MD 21702, USA; suzanne.e.wollen.ctr@mail.mil (S.E.W.); farooq.nasar.ctr@mail.mil (F.N.); joshua.d.shamblin.civ@mail.mil (J.D.S.); kern.steven0@gmail.com (S.J.K.); anna.honko@nih.gov (A.N.H.); william.d.pratt.civ@mail.mil (W.D.P.); margaret.l.pitt.civ@mail.mil (M.L.M.P.); 2 Pathology Division, U.S. Army Medical Research Institute of Infectious Diseases, 1425 Porter St., Fort Detrick, MD 21702, USA; jeremy.j.bearss.mil@mail.mil (J.J.B.); michelle.a.jefferson.mil@mail.mil (M.A.J.); taylor.b.chance.mil@mail.mil (T.B.C.); 3 Molecular and Translational Sciences, U.S. Army Medical Research Institute of Infectious Diseases, 1425 Porter St., Fort Detrick, MD 21702, USA; jeffery.r.kugelman.mil@mail.mil (J.R.K.); jason.t.ladner.ctr@mail.mil (J.T.L.); gustavo.f.palacios.ctr@mail.mil (G.F.P.); 4 Battelle Memorial Institute, 505 King Ave., Columbus, OH 43201, USA; kobsd@battelle.org (D.J.K.); wendingm@battelle.org (M.Q.S.W.); SabourinC@battelle.org (C.L.S.)

**Keywords:** Ebola virus, Kikwit, filovirus, nonhuman primate, glycoprotein, RNA editing, pathogenesis, animal model, vaccine, therapeutic

## Abstract

Recent experimentation with the variants of the Ebola virus that differ in the glycoprotein’s poly-uridine site, which dictates the form of glycoprotein produced through a transcriptional stutter, has resulted in questions regarding the pathogenicity and lethality of the stocks used to develop products currently undergoing human clinical trials to combat the disease. In order to address these concerns and prevent the delay of these critical research programs, we designed an experiment that permitted us to intramuscularly challenge statistically significant numbers of naïve and vaccinated cynomolgus macaques with either a 7U or 8U variant of the Ebola virus, Kikwit isolate. In naïve animals, no difference in survivorship was observed; however, there was a significant delay in the disease course between the two groups. Significant differences were also observed in time-of-fever, serum chemistry, and hematology. In vaccinated animals, there was no statistical difference in survivorship between either challenge groups, with two succumbing in the 7U group compared to 1 in the 8U challenge group. In summary, survivorship was not affected, but the Ebola virus disease course in nonhuman primates is temporally influenced by glycoprotein poly-U editing site populations.

## 1. Introduction

The filoviral genus *Ebolavirus* has five members, Bundibugyo virus (BDBV), Ebola virus (EBOV), Reston virus (RESTV), Sudan virus (SUDV), and Taï Forest virus (TAFV). EBOV, the only member of the species *Zaire ebolavirus*, is a hemorrhagic fever virus and, next to BDBV, SUDV, and TAFV, is a causative agent of Ebola virus disease (EVD), which is associated with ≈40% lethality. EBOV has a negative single strand RNA genome of approximately 19,000 nucleotides containing 7 genes (NP, VP35, VP40, GP, VP30, VP24, L) and produces long filamentous and/or morphologically heterogeneous virions. Its genome maximizes product output through editing of the glycoprotein RNA to produce three different products, soluble GP (sGP), small soluble glycoprotein (ssGP), and full-length glycoprotein (GP_1,2_), which each decorate the exterior of the enveloped virion and mediate viral entry into the host cell, in addition to its association with EBOV pathogenesis such as endothelial dysfunction and cytotoxicity [1,2,3]. sGP is secreted from infected cells and has been associated with antigenic subversion, a process by which sGP may act as a decoy for host immune response by acting as an antibody decoy [4]. There is no known function for ssGP. Wild-type virus GP gene sequences, isolated from human samples, consist of a 7-uridylyl stretch, poly-U site in the glycoprotein sequence that produces sGP as a major product (~75% of transcripts). A minority population of transcripts are edited (~25%) and contain an extra adenosine incorporated through the stuttering of the transcriptional machinery, thus encoding full length GP_1,2_ [2,3,5].

Recent data have demonstrated that there are three significant mutations in EBOV GP gene sequence may arise in the viral population after passage in cell culture; the most dramatic change is associated with an indel at the poly-U site, position 6918 to 6924, where an extra U has been inserted, bringing the total U content to 7 + 1 = 8 [5]. This change occurs within 24 post-infection and, as a result, flips the normal production ratios of sGP:GP_1,2_ such that GP_1,2_ is now the dominant product [2,5]. The serially cell-culture-passaged virus with 8 uridines at the GP poly-U site demonstrated enhanced growth kinetics *vs.* the virus with only 7 uridines, such as a large plaque phenotype and higher titers [6]. Interestingly, when this cell-culture-adapted virus (termed 8U EBOV hereon) is used to infect guinea pigs, the viral population quickly reverts back to wild-type population ratios at the poly-U site (termed 7U EBOV hereon) as well as the sGP:GP_1,2_ ratios [6]. This reversion was also observed when the 8U variant was used to infect NHPs [5]. Guinea pigs infected with the 8U EBOV also experienced a delay in death [6]. A comparison experiment between 7U and 8U has not been conducted in NHPs for EBOV, but Alfson *et al.,* compared the two for SUDV and found no differences [7]. The two other mutations that arise in the EBOV viral population after passage *in vitro* were non-synonymous mutations at position 6179 (E47D) in the glycoprotein and at position 10833 (R163K) in VP24.

Since the start of the largest EBOV outbreak on record with over 26,000 cases as of 22 April 2015 [8], numerous therapeutic and vaccine platforms have been implemented in human clinical trials, have been “compassionate[ly] use[d]” for off-label approved drugs, have expanded access of investigational drugs, and have been implemented in non-clinical animal research [9,10,11,12,13,14,15,16,17,18,19,20,21,22]. The path forward to licensing either therapeutic or vaccine countermeasures against EBOV will likely require evaluation under the FDA “Animal Rule”. Under this provision [23], efficacy of the countermeasure in question must be shown in a relevant and “sufficiently well characterized animal model for predicting response in humans”. Nonhuman primates (NHPs), specifically macaques, have been used for the past 40 years as the model for EVD and EBOV pathogenesis in humans [21,24,25,26,27,28,29,30,31,32,33,34,35,36,37,38]. However, there is no data available from NHP models regarding the effects of challenge with either the 7U EBOV or 8U EBOV. The challenge of NHPs with the cell-culture-derived virus 8U EBOV may result in either a less lethal model or longer time for the onset of the disease due to the lack of antigenic subversion normally associated with the wild-type 7U EBOV. Therapeutics, such as the ZMapp antibody cocktail, target EBOV GP and as such may have different efficacies in critical path NHP challenges with a 7U EBOV compared to 8U EBOV. Current vaccine platforms in clinical trials, such as VSVΔG and ChAd3, rely on EBOV GP expression to induce an immune response and protect against EVD. If one attempts to compare vaccine platforms performed by Study Team X (challenging with 7U virus) and Study Team Y (challenging with 8U virus), there may be inherent differences in GP and disease course that make one set of challenges a “higher bar” for the vaccine platform to overcome EBOV. These challenge stock differences would significantly impact the conclusions drawn from the data should there truly be a difference between the 7U and 8U EBOV challenge materials.

A direct comparison of the disease caused by EBOV stocks containing 7U and 8U dominant populations was required to determine if the 7U EBOV is more pathogenic than its 8U counterpart in naïve macaques. This empirical comparison between 7U and 8U EBOV was also conducted in the context of a potential EBOV GP-based vaccine candidate. To accomplish this direct, data-driven comparison, a statistically significant number of naïve cynomolgus macaques were challenged intramuscularly with either 7U or 8U EBOV. Disease course was evaluated by study staff blinded to the identity of challenge stock. The data presented highlight selection pressures exerted by dynamic host-pathogen interactions enabling selection of virus populations with fitness tradeoffs. Here we investigated the 7U and 8U, disease course, pathogenicity, and viral population dynamics in both naïve and vaccinated NHPs.

## 2. Materials and Methods

### 2.1. Animals

Research was conducted under an IACUC-approved animal protocol at the United States Army Medical Research Institute of Infectious Diseases (USAMRIID). This protocol complied with the Animal Welfare Act, PHS Policy, and other Federal statutes and regulations relating to animals and experiments involving animals. The facility where this research was conducted is accredited by the Association for Assessment and Accreditation of Laboratory Animal Care, International and adheres to principles stated in the Guide for the Care and Use of Laboratory Animals, National Research Council, 2011. All experiments were conducted in USAMRIID’s ABSL-4 laboratory.

### 2.2. Study Design

Forty-four cynomolgus macaques (*Macaca fascicularis*), of Chinese origin, equally balanced by sex, were randomized, coded for blinding (both challenge material identity and vaccination identity), and randomized into naïve control and vaccinate groups for both the 7U and 8U challenge materials. The 7U EBOV group consisted of 14 naïve animals and 8 vaccinates. The 8U EBOV group mirrored the 7U challenge group with 14 naïve animals and 8 vaccinates. Phlebotomies via peripheral vein and physical examinations were conducted under anesthesia on days: pre-challenge, 0, 3, 6, 10, 14, 21, 28, and terminal.

Three types of survival statistical analyses were performed: A Kaplan-meier analysis with a log-rank test of homogeneity was performed to test for overall survival differences between groups. The percentage of survival was compared using Fisher’s exact test for the direct comparisons between the two groups. Time of death was compared between groups using the animals that succumbed.

Secondary assays (*i.e.*, chemistry, hematology, and qRT-PCR data) were statistically analyzed via Box-Cox transformations, followed by modeling using a mixed model with repeated measures. Changes from baseline were assessed for each group through model-adjusted group mean comparison to zero at each time point. Group comparisons were made similarly via pairwise model-adjusted mean comparison. Multiple testing was corrected using permutation.

### 2.3. Vaccination

One goal of these experiments was to assess the difference between 7U and 8U EBOV in the presence of a vaccine; for this purpose, we utilized a proprietary, research grade vaccine. A total of 16 animals were vaccinated with GP-based vaccine. To confirm a successful and specific immune response to EBOV from the vaccination, antibody titers specific for EBOV GP_1,2_ were assessed via ELISA, as previously described, at weekly intervals post-vaccination [14]. The EBOV exposure occurred one week after the vaccination schedule was completed.

### 2.4. Challenge

All forty-four cynomolgus macaques were intramuscularly exposed to Ebola virus/H.sapiens-tc/COD/1995/Kikwit-9510621 (EBOV) at a target dose of 1000 pfu 7U EBOV (USAMRIID challenge stock “R4415”; GenBank # KT762962) or 8U EBOV (stock “R4368”; GenBank # KT582109) on Day 0. Both 7U and 8U EBOV challenge materials were analyzed via high-throughput Illumina MiSeq deep sequencing to 100% coverage, 150 bp paired-end format as described previously [5], prior to challenge for alignment to the Ebola virus/H.sapiens-tc/COD/1995/Kikwit-9510621 stock “134” reference sequence (GenBank # AY354458) as well as percent 7U *vs.* 8U composition. Challenge dose was determined via neutral red plaque assay with an agarose overlay as described previously [39]. Particle to pfu ratio was determined using neutral red plaque assay [40] and electron microscopy similar to Alfson *et al.* [8,40].

### 2.5. Telemetry

Eight naïve cynomolgus macaques in each challenge group, a total of 16 animals, were implanted with the T2J temperature and activity radio telemetry devices (Konigsberg Instruments Pasadena, CA, USA), sutured into the right inside abdominal wall at least three weeks prior to challenge. Following acclimation to the ABSL-4 laboratory environment, baseline data was established through a 5-day data acquisition period prior to challenge (Day −5 to 0 post-exposure) to account for normal diurnal rhythm and individual variability. All analog data signals were converted to digital data and analyzed via Notocord-hem Evolution software (Version 4.3.0.43, Notocord Inc., Newark, NJ, USA). Continuous data was reduced to 30-min averages across the 24 h day and exported for statistical analysis compared to the baseline data for each individual animal. Baseline telemetry data for statistics were fit using a Bayesian multiple-component autoregressive cosinor model with t-distributed error. Samples were drawn from the posteriors using Hamiltonian Monte Carlo, as implemented in Stan, using four chains, each with a warmup of 2500 draws, followed by 12,500 samples for a total of 50,000 posterior points.

### 2.6. Animal Observations and Euthanasia

Animals were evaluated daily by study personnel for signs of illness (responsiveness, telemetry temperatures, cough, edema, rash, bleeding, motor function [41]). Other observations such as biscuit/fruit consumption, condition of stool, and urine output were also documented, but not calculated for euthanasia criteria. Animals were observed approximately every 4.5 h upon the first signs of EVD with a 6 h rest from midnight to 0600 h. Decisions for humane endpoints were prescribed by a previously defined algorithm [41]. All euthanasia events occurred via intra-cardiac administration of a pentobarbital-based euthanasia solution while the animals were under deep anesthesia.

### 2.7. Chemistry

For serum chemistries, whole blood was collected into Z Serum Clot Activator Greiner Vacuette tubes (Greiner Bio-One, Monroe, NC, USA). Tubes were gently inverted by hand to ensure adequate mixing and placed upright. Tubes were allowed to clot for 30 min and then centrifuged at 1800× *g* for 10 min at ambient temperature. Serum was separated from the clot within 2 h of collection and the required volume of serum was removed for chemistry analysis using a Piccolo Point-Of-Care Analyzer (Abaxis, Union City, CA, USA) and Piccolo General Chemistry 13 panel.

### 2.8. Hematology

Whole blood was collected into K3 EDTA Greiner Vacuette blood tubes, which were then gently inverted by hand to ensure adequate mixing. Hematology was performed on the ADVIA 120 (Siemens Healthcare, Malvern, PA, USA). Following the completion of the CBC, white blood cell differential, and reticulocyte analyses, the remaining blood was centrifuged to separate the plasma in a centrifuge set at 2500× *g* for 10 min at ambient temperature. Centrifugation occurred within 6 h of collection.

### 2.9. qRT-PCR and Sequencing

A 100 µL volume of EDTA plasma was added to 300 µL of TriReagent LS (Sigma, St. Louis, MO, USA) in preparation for qRT-PCR. Inactivated samples were extracted and eluted with AVE Buffer (Qiagen, Valencia, CA, USA) using a QIAamp Viral RNA Mini Kit (Qiagen). All samples used in qRT-PCR on an Applied Biosystems 7500 Fast Dx Real-Time PCR instrument (Life Technologies, Grand Island, NY, USA). The RT-PCR reaction was performed using the SuperScript II One-Step RT-PCR System (Life Technologies, 12 Grand Island, NY, USA), with additional MgSO_4_ added to a final concentration of 3.0 mM. Samples were run in triplicate using a 5 µL volume, with the average of the triplicates being multiplied by 200 to obtain genomes equivalents mL^−1^, and then multiplied by a dilution factor of 4 for the final reported value. The sequence of the primer and probes for the EBOV glycoprotein are described below. The genomic equivalents were determined using a synthetic RNA standard curve of known concentration.

Forward primer: 5' - TTT TCA ATC CTC AAC CgT AAg gC - 3'
Reverse primer: 5' - CAg TCC ggT CCC AgA ATg Tg - 3'
Probe: 6FAM - CAT gTg CCg CCC CAT CgC TgC - TAMRA

Viral population sequencing was performed on the Illumina platform using anti-genomic amplicon sequencing as previously described with alignments performed using AY354458 as a reference [5].

### 2.10. Necropsy

Necropsies were conducted at BSL-4 by a board certified veterinary pathologist on all animals in this study. The following tissues were collected for histopathology on the animals: haired skin with tattoo, haired skin with rash (if present), thigh muscle (injection site and contralateral from injection site), axillary lymph node, inguinal lymph nodes, tracheobronchial lymph node, spleen, liver, gallbladder, kidneys, adrenal glands, and lungs.

### 2.11. Histology

Tissues were fixed by immersion in 10% neutral buffered formalin for a minimum of 21 days prior to removal from BSL-4 and processing for histology. The tissue samples were trimmed, processed, and embedded in paraffin. Sections of the paraffin-embedded tissues 5 µm thick were cut for histology. After routine hematoxylin and eosin (H & E) staining, slides were evaluated microscopically by a board certified veterinary pathologist.

## 3. Results

### 3.1. Challenge

Each challenge virus stock was sequenced prior to challenge to confirm the majority population with regard to the poly-U site in the EBOV GP gene. Both stocks were originally obtained from the same clinical specimen and differed only in the number of passages in cell culture. The 7U EBOV was 92.8% 7U at the poly-U site of GP, while the 8U EBOV poly-U site in GP was 92.6% 8U [5]. On Day 0, all animals were challenged intramuscularly in the right thigh with a 1000 pfu target dose of either 7U or 8U EBOV. The 7U challenge group received 1249 ± 355 pfu per animal and the 8U challenge group received 1088 ± 54 pfu per animal. Particle to pfu ratios were 1.4 × 10^3^ and 6.8 × 10^3^ for the 7U and 8U stocks, respectively. The less than five-fold difference between the two would not be consistent with the ≥1log particle to pfu differences that resulted in differing EVD previously observed [40].

### 3.2. Disease Course

After challenge, all animals remained free from signs of Ebola virus disease (EVD) until Day 5 post-exposure. On Day 5 post-exposure, 13 of 14 naïve NHPs in the 7U EBOV group (Table S1) exhibited decreased activity compared to 7 of 14 naïve NHPs in the 8U challenge group (Table S2). This trend was similar in the development of anorexia with 7 of 14 naïve NHPs in the 7U challenge group having reduced appetite on Day 5 post-exposure compared to only one of the naïve NHPs showing decreased appetite on Day 5 post-exposure for the 8U EBOV naïve NHPs. The onset of typical macular EVD rash occurred earlier in the naïve 7U challenge group, with 4 of 14 NHPs showing rashes on Day 5 post-exposure. None of the naïve 8U EBOV NHPs had rashes on Day 5 post-exposure, but 7 of 14 showed signs of rash beginning on Day 6 post-exposure. Aside from these differences in time of onset of disease signs, both challenge groups’ naïve NHPs exhibited the same characteristic signs of EVD to a similar degree.

### 3.3. Survival and Time of Endpoint

**Figure 1:**
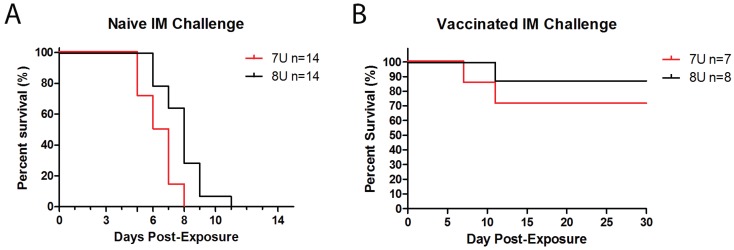
Survival. (**A**) Fourteen NHPs in each challenge group, 7U (**Red**) or 8U (**Black**), were exposed to a target dose of 1000 pfu EBOV on Day 0 post-exposure. The percentage of survival was documented per day post-exposure; (**B**) Groups of 8 NHPs were vaccinated with a proprietary EBOV-GP based vaccine and then exposed to a target dose of 1000 pfu EBOV on Day 0 post-exposure. The percentage of survival was documented per day post-exposure.

There was a significant difference in the time of death between the 7U and 8U naïve challenge groups (Figure 1A; *p* = 0.0016). The 7U EBOV group reached their terminal time points on average 6.46 days after challenge, which was 1.52 days prior to the 8U EBOV group that averaged 7.98 days post-exposure. Remarkably, the differences in endpoint between the 7U and 8U EBOV naïve challenge did not translate to a statistically significant difference for vaccinated animals. The two vaccinates that succumbed to EVD in the 7U challenge group had endpoints of 7 and 11 days post-exposure. The analysis of the 7U EBOV vaccinate group was comprised of 7 animals instead of 8 due to the exclusion of one animals as a result of humane endpoint based on veterinarian concerns regarding behavioral issues. The 8U group had a single vaccinate succumb to EVD on Day 11 post-exposure.

There was no difference in survivorship between the naïve NHPs for both the 7U and 8U EBOV challenge groups. All 14 naïve NHPs for both the 7U and 8U challenge groups succumbed to EVD (Figure 1A). Survivors were observed in the vaccinated groups, with 5 of 7 vaccinates surviving the 7U challenge (one euthanized for humane reasons and excluded from analysis) and 7 of 8 surviving the 8U EBOV challenge (Figure 1B). Despite the different numbers of vaccinates surviving each respective challenge, there was no statistically significant difference in survivorship between either the 7U or 8U challenge groups among all vaccinated animals.

### 3.4. Vaccination

**Figure 2:**
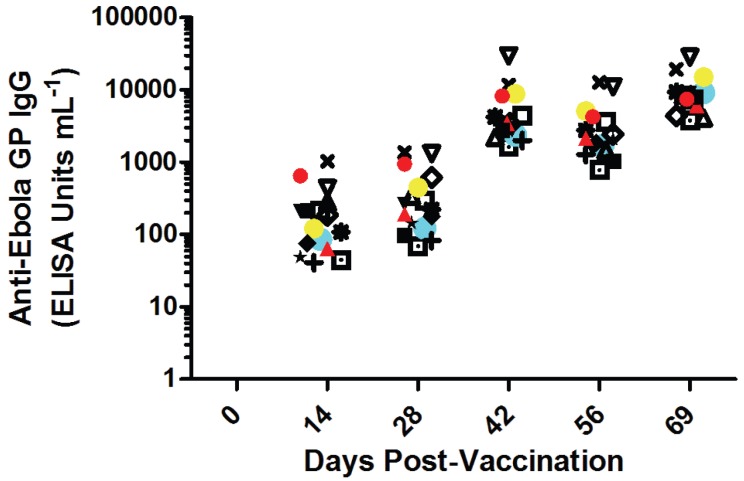
Immunogenicity of EBOV GP-based vaccination prior to challenge. Sixteen monkeys were vaccinated on Day 0 and boosted on Days 28 and 56. Each animal’s anti-Ebola virus glycoprotein titers are shown as unique symbols at each time point. Animals were challenged with EBOV on Day 72 post-vaccination. Red symbols are animals that succumbed to 7U challenge stock (*n* = 2). Blue symbols are animals that succumbed to 8U challenge stock (*n* = 1). Yellow symbols represent the *n* = 1 animal euthanized for humane reasons un-attributable to Ebola virus exposure. None of the data points were significantly different from each other at each time point.

Sixteen cynomolgus macaques were vaccinated with a proprietary vaccine based on EBOV GP. Each animal’s individual antibody titers specific for EBOV GP are shown in Figure 2. Antibody titers one week prior to challenge were as high as 2.8 × 10^4^ ELISA units mL^−1^ and as low as 3.7 × 10^3^ ELISA units mL^−1^. There was no significant difference between vaccinates slated for challenge with 7U EBOV compared to those of 8U EBOV, which suggests that there was no pre-challenge bias between the 7U and 8U groups’ immune response to vaccination that might affect survivorship.

### 3.5. Telemetry

**Figure 3:**
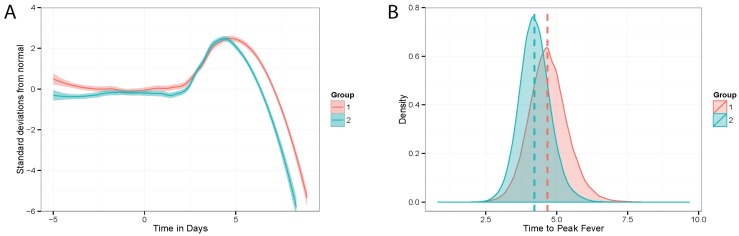
Fever among naïve animals implanted with radio-telemetry devices. (**A**) LOESS curve representing the estimate of average normalized values across each treatment group (Group 1 = 8U **red** (*n* = 8); Group 2 = 7U **blue** (*n* = 8)); (**B**) Posterior predictive distributions of peak fever times across treatment groups (Group 1 = 8U **red** (*n* = 8); Group 2 = 7U **blue** (*n* = 8)).

The telemetry device implantation of naïve controls (*n* = 8 per challenge isolate) permitted the real-time acquisition of temperature data throughout the study. The overall trends in the telemetry data are presented in Figure 3A, which shows the difference from the normal values as measured in standard deviations. A disruption of diurnal rhythm, a departure from zero deviation compared to the pre-exposure data, was detected as early as 2 days post-exposure. The mean time of peak fever, defined as the maximum point prior to final descent and succumbing to EVD, was 4.7 days post-exposure for the 8U group and 4.23 post-exposure for the 7U group (Figure 3A). The probability of the difference in peak fever times beginning within the region of practical equivalence of [−0.5, 0.5] day is 0.5372, suggesting that it is unfeasible to accept the null hypothesis that the time of peak fever is the same between the 8U and 7U naïve groups, within a margin of 12 h. Rather, there is some evidence that the time of peak fever is greater for the 7U group, with a probability of 0.9465 (Figure 3B).

### 3.6. Chemistry

**Figure 4:**
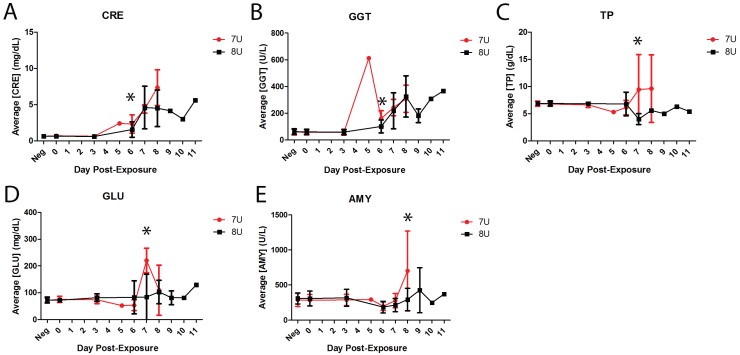
Average serum chemistry in naïve challenge groups. All error bars represent standard deviation. Asterisks indicate statistically significant differences between the two groups (7U, *n* = 14, **red**; 8U, *n* = 14, **black**). (**A**) Average concentration of creatinine in serum for naïve NHPs shown in mg/dL; (**B**) Average concentration of gamma-glutamyl transpeptidase for naïve NHPs in serum shown in U/L; (**C**) Average concentration of total protein in serum for naïve NHPs shown in g/dL; (**D**) Average concentration of glucose in serum for naïve NHPs shown in mg/dL; (**E**) Average concentration of amylase in serum for naïve NHPs shown in U/L.

**Figure 5:**
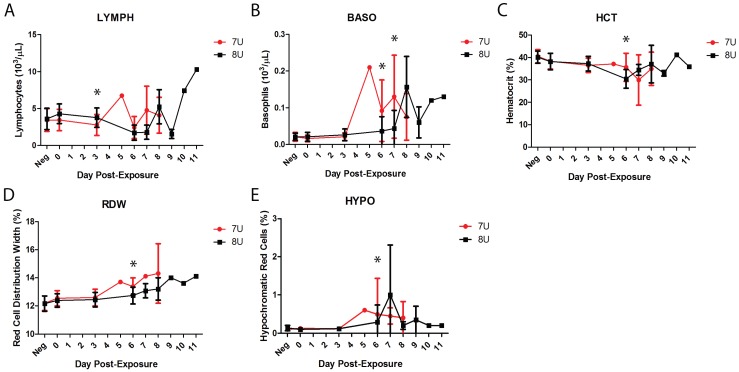
Average hematology values in naïve challenge groups. All error bars represent standard deviation. Asterisks indicate statistically significant differences between the two groups (7U, *n* = 14, **red**; 8U, *n* = 14, **black**). (**A**) Average concentration of lymphocytes in whole blood for naïve NHPs shown in 1000 cell per µL; (**B**) Average basophils in whole blood for naïve NHPs shown in 1000 cells per µL; (**C**) Average hematocrit of naïve NHPs by percent; (**D**) Average red cell distribution width for unvaccinated control NHPs shown as percent; (**E**) Average percent hypochromatic red cells in naïve NHP whole blood.

Serum chemistry was analyzed via basic Chem 13 panel at each phlebotomy. While all parameters were consistent with EVD (*i.e.*, typical azotemia and spike in liver enzymes) [42], there were 5 analytes that were statistically significant in difference between average group values at a minimum of one time point. The earliest of these statistically significant differences occurred on Day 6 post-exposure where both creatinine (CRE) and gamma-glutamyl transpeptidase (GGT) were significantly higher in the 7U EBOV group compared to the 8U EBOV naïve controls, *p* = 0.0481 and *p* = 0.0036 respectively (Figure 4A,B). On Day 7, post-exposure both total protein (TP, Figure 4C) and glucose (GLU, Figure 4D) were significantly higher in the 7U challenge group, *p* = 0.0001 and *p* = 0.0041 respectively. The latest significant difference between the two challenge groups occurred on Day 8 post-exposure, where concentrations of amylase (AMY, Figure 5E) in the serum were significantly higher in the 7U group, *p* = 0.0054.

### 3.7. Hematology

Whole blood drawn at each scheduled phlebotomy was subjected to complete blood count (CBC), white blood cell differential, and reticulocyte analysis. Of the 27 measured and derived parameters analyzed, five categories were significantly different between the 7U and 8U EBOV challenge groups on at least one day post-exposure. On Day 3 post-exposure the concentration of circulating lymphocytes (LYMPH, Figure 5A) was significantly lower in the 7U EBOV naïve controls compared to the 8U EBOV naïve controls, *p* = 0.0448. The majority of the statistically significant differences between the two challenge groups occurred on Day 6 post-exposure when the 7U naïve controls had significantly higher values for basophils (BASO, Figure 5B), hematocrit (HCT, Figure 5C), red cell distribution width (RDW, Figure 5D), and hypochromatic red cells (HYPO, Figure 5E), *p* = 0.0264, *p* = 0.0311, *p* = 0.0213, and *p* = 0.0348 respectively. The latest of the significant differences in hematology between the two challenge groups was on Day 7 post-exposure, when basophils were again significantly higher in the 7U EBOV group, *p* = 0.0481 (Figure 5B).

### 3.8. Pathology

The lesions noted at gross necropsy for all naïve NHPs were consistent with the reported course of EVD in NHPs, with no noticeable differences between the 7U and 8U naïve challenge groups. The most consistent and significant of these gross necropsy findings were a macular rash on the skin, most often involving the face, inguinal, and axillary areas; pale discoloration of the liver with increased friability of the liver parynchyma; enlarged, turgid spleen; enlarged, hemorrhagic lymph nodes, most notable in the inguinal and axillary nodes; and pale discoloration of the kidneys. All the surviving vaccinates, including the animal from the 7U group that was euthanized for ethical reasons, lacked these characteristic EVD lesions during gross necropsy.

Histologic lesions in tissues taken from the untreated controls were also consistent with known EVD pathology (examples shown in Figure S1). All animals succumbing to EVD demonstrated lymphocyte necrosis (lymphocytolysis) and subsequent depletion of lymphoid tissue of the splenic white pulp and one or more of the examined lymph nodes (Figure S1A,C). Additionally, the histological analyses showed random foci of hepatocyte degeneration and necrosis in the liver for all succumbing NHPs, with infrequent eosinophilic cytoplasmic inclusion bodies in hepatocytes and fibrin thrombi in hepatic vasculature (Figure S1B). Fibrin thrombi were found in the medulla of the kidneys and in the cortex of the adrenal glands of all succumbing NHPs (Figure S1D). No significant differences were observed between the 7U and 8U challenge groups during the histological analyses.

### 3.9. Circulating Viral Genome Equivalents

**Figure 6:**
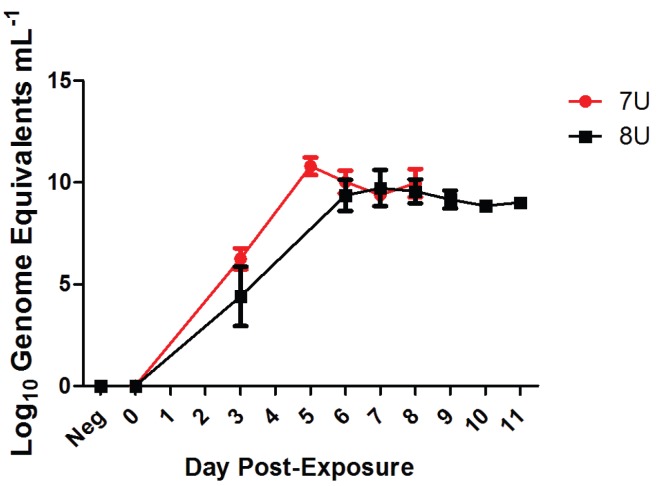
Circulating viral genome equivalents per mL of plasma. Average concentration of circulating viral genome equivalents per mL of plasma across both challenge groups for the naïve NHPs (7U, *n* = 14, **red**; 8U, *n* = 14, **black**). Error bars represent standard deviation.

At each scheduled phlebotomy and at terminal time points, plasma samples were analyzed via qRT-PCR for the presence of circulating viral RNA. Viral genetic material was found in the plasma as early as Day 3 post-exposure and peaked by Day 6 post-exposure in the naïve animals (Figure 6). The 7U challenge group had two animals succumb to EVD on Day 5 post-exposure, but, since this was not a scheduled phlebotomy, no comparison was made to the 8U challenge group, which had no terminal events on Day 5 post-exposure. Once the circulating genome equivalent concentrations peaked by Day 6, the levels remained consistent between 7.19–10 log_10_ genome equivalents mL^−1^ until each animal succumbed to EVD (Figure 6). There were no significant differences between the 7U and 8U EBOV naïve animals.

The vaccinated animals had no viral genome equivalents found at Day 3 post-exposure in either 7U or 8U EBOV group. Most of the animals in either the 7U or 8U challenge group had near sterile immunity throughout the challenge course of the experiment (*i.e.*, viral genomic equivalent levels below the limit of detection). Only two vaccinated NHPs had quantifiable viral titers in the 8U challenge group, with one succumbing (Day 11) and the other surviving challenge (Table 1). The 7U challenge group had four vaccinated animals with detectable levels of circulating viral genome equivalents, with two NHPs surviving challenge and two succumbing to EVD (Day 7 and Day 11). The vaccinated animals that survived EBOV challenge group in either group did not reach concentrations of greater than 5.99 log_10_ genome equivalents mL^−1^.

**Table 1:** Vaccinated NHP circulating viral genome equivalents. Each quantifiable circulating genome concentration (log_10_) for all vaccinated monkeys in both 7U EBOV and 8U EBOV challenge groups is listed by day. Dashes represent time points for which there was no quantifiable data. Asterisks indicate a terminal event.

	7U (*n* = 4 Quantifiable)		8U (*n* = 2 Quantifiable)	
	Survived	Succumbed	Survived	Succumbed
Day 6	5.96, 5.44	7.19, 10.31	-	5.23
Day 7	-	11.09 *	-	-
Day 10	5.99	7.46	5.82	10.53
Day 11	-	7.42 *	-	10.40 *

### 3.10. Viral Population Dynamics

**Figure 7:**
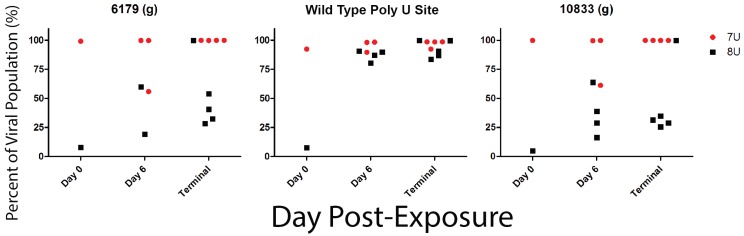
Viral Population Dynamics. A subset of whole blood from each cohort was sequenced to characterize changes in the whole genome over the course of infection. The number at the top of the chart indicates the sequence position, in relation to AY354458 as a reference, and the letter in parentheses represents the base at that given position. Both 6179 and 6925 positions within GP and 10833 are located in VP24. Red circles are monkeys from the 7U challenge group, while black squares represent the 8U challenge group.

As mentioned previously, the input viral population genetics of the poly-U stutter site in GP were confirmed via sequencing prior to challenge, which revealed the 7U challenge stock to be 92.8% 7U and the 8U challenge stock to be 92.6% 8U. Position 6179 and 10833 were identified as other changes in the challenge material where the consensus sequence of the 7U and the 8U variants differed; these changes are consistent with previous reports of cell culture passage of EBOV [5]. Samples were sequenced throughout the course of the study at each scheduled phlebotomy; however, there were no samples reliably sequenced before Day 6 post-exposure due to potential amplification bias. Reliable sequences were obtained on Day 6 post-exposure, at which time a clear reversion of the 8U challenge stock to 7U was evident (Figure 7, 6925 (a)). The reversion was dramatic at position 6925, the poly-U site, with the percentage of the viral population changing approximately 62-fold, from 1.5% to 92.6% of the total population. All of the 8U challenged animals sequenced demonstrated this near-complete reversion to 7U at 6925, which remained consistent until the endpoint of the experiment. The other reversal changes observed in the 8U challenge stock, 6179 and 10833, compared to the 7U were also tracked during the course of infection. Those animals challenged with the 8U variant experienced slight drift in the populations at both positions, but the two challenge groups clustered together at each time point. The clustering of 7U and 8U populations and the maintenance of these clusters throughout challenge, combined with the limited change in percent of total population, would suggest that the main selection pressure is excerpted into 6925, the poly-U editing site.

## 4. Discussion

While it has been performed for SUDV, there are no published reports comparing the potential difference in pathogenesis between 7U and 8U EBOV in NHPs [7]. One may anecdotally compare what data are available for NHP experiments that were identified as using either 7U or 8U challenge stocks; however, this type of comparison is difficult due to the lack of sequence data, or viral pedigree, presented for historical studies. In fact, only a very few recent publications identify the virus stock as 7U or 8U after first reported by Volchkov *et al.* [2]. A recent study by Mire *et al.* listed the time of death of naïve cynomolgus macaques challenged with a 7U EBOV stock at Days 7 and 8 post-exposure [13]. Another recent study in rhesus macaques by Thi *et al.* had times of death between Day 8 and Day 9 post-exposure [11]. These timeframes are consistent with previous data for macaque models of EVD with cynomolgus macaques succumbing on average to EVD between Days 6-9 post-exposure and rhesus macaques between Days 7–10 post-exposure [21,24,25,26,27,28,29,30,31,32,33,34,35,36,37,38]. Further complicating, if not preventing this method of comparison, are the different euthanasia criteria that may be implemented between institutes that impact time-of-euthanasia decisions [41]. Standardization of euthanasia criteria between study sites and teams within the same site would aide with this type of comparison in the future.

The 7U and 8U EBOV data presented herein had time-of-euthanasia events that were consistent with the averages reported in the literature for naïve cynomolgus macaques, ranging from 5–9 days post-exposure between the two groups [21,24,25,26,27,28,29,30,31,32,33,34,35,36,37,38]. This time frame is unremarkable when considering each group individually, which may be a confounding factor in determining 8U EBOV pathogenicity. However, this study was run as a blinded, head-to-head comparison of the disease course caused by exposure to either the 7U EBOV or 8U EBOV challenge stocks, which demonstrated that naïve monkeys in the 7U EBOV challenge group (*n* = 14) succumbed to infection 1.53 days on average prior to naïve monkeys in the 8U EBOV challenge group (*n* = 14), (*p* = 0.0016). During the prospective design of this study, considerations were made to prioritize statistical requirements, as well as blinding and randomization, to ensure a valuable outcome at the conclusion of the experiment. In order to achieve appropriate levels of statistical significance and detect a difference of ≥1.25 days between the mean times of euthanasia of the challenge groups, minimum cohort sizes of 14 NHP were each required for each challenge stock group (7U *vs.* 8U). Current summaries of ongoing clinical trials demonstrate the importance of generating well controlled, blinded, and statistically significant populations for evaluating countermeasure efficacy in humans, a principle that will need to be carried over to Animal Rule research [23,43,44].

The sequence data acquired in this experiment were consistent with previous data from *in vivo* experiments where an 8U EBOV challenge stock quickly reverted to 7U [6,45]. Since both the 7U and 8U EBOV stocks were not 100% pure populations as one might produce through the use of clones, the mechanism of change from 8U back to 7U cannot be answered from these experiments. However, the sequencing data from these experiments demonstrated that the 8U population reverted to 7U at the poly-U editing site shortly after challenge. The two other reversal changes observed in GP and VP24 were minor compared to the >90% change in the poly-U site by Day 6 post-exposure and are consistent with previous data (<2-fold change by Day 5) [5]. These other sequence discrepancies, at positions 6179 and 10833, are unlikely to be attributable to the significant difference in disease courses since these changes were limited and appear to be compensatory in nature. Additionally, NHPs were already experiencing differences in disease course, such as fever, prior to Day 6, suggesting that the genetic changes associated with the delayed disease course occurred earlier. Furthermore, the change in VP24 sequence has not yet been attributed to any phenotypic outcome. In structural terms, it is postulated to have an insignificant effect on the structure of the protein [5]. Taken together these data suggest that the difference at the poly-U editing site is the likely factor for the delayed disease course among naïve 8U challenged NHPs.

The potential mechanism(s) behind the differences between challenge groups could be explained by the ratio of the sGP:GP_1,2_ protein. High levels of GP_1,2_, such as those associated with 8U EBOV, have shown significant cytotoxicity, which may result in local damage around the area of parenteral administration and thus recruit larger numbers of susceptible dendritic cells or macrophages early on [46,47]. These same cell types are typically associated with high levels of proinflammatory cytokines and may explain why the naïve NHPs in the 8U EBOV challenge group experienced fever earlier than the 7U EBOV group [48]. This same process may also be exacerbated by the effects of shed GP, a soluble form of surface GP released from the cells by the enzyme TACE/ADAM-17, which mediates the release of proinflammatory cytokines and the upregulation of costimulatory molecules on antigen presenting cells and B cells [49,50]. The process of antigenic subversion might be attributable to the more rapid onset of disease caused by 7U EBOV [4]. It is likely that the increased levels of sGP that are produced immediately after infection masked the 7U EBOV infection, and, when combined with the lack of cytotoxic effects associated with the over expression of GP_1,2_, resulted in the earlier time frame of disease for 7U EBOV challenge (*i.e.*, increased ability of the host immune system to defend against 8U EBOV) [46,49]. These data support recent findings that surface GP levels are controlled through GP RNA editing, and this regulation plays a distinct role in pathogenicity [51].

It is important to note that there was no significant difference in endpoint between 7U EBOV and 8U EBOV challenges. The final disposition of each NHP was consistent with EVD in cynomolgus macaques [52]. Gross pathology and histology also revealed no departure from typical EVD historically reported in the literature [53]. Guinea pig studies with clones engineered to express either 7U or 8U have confirmed that either challenge results in fatal EVD with the 8U rapidly reverting to 7U [51,54]. Given the lack of differences between the end stage disease and the rapid return back to a predominantly 7U genotype, the data presented herein support the conclusion that an 8U challenge is attenuated compared to a 7U challenge, but only in the disease course and not in terms of lethality, especially considering that all animals had reverted back to the 7U majority population by the time of euthanasia. The minority 8U EBOV population maintenance *in vivo* is likely a balance of immune system selection resulting in a mechanism for regulating GP expression.

The difference in time-of-euthanasia presented herein, combined with the recommendations of the FDA’s Animal Rule guidance to use the challenge agent most similar to the etiological agent in human disease, clearly support the selection of 7U EBOV in NHP challenge studies, since the 8U EBOV dominant population is a cell-culture artifact [5,23]. The use of 7U EBOV for challenge becomes more critical when taken in the context of therapeutic evaluation, where 8U challenge could artificially lengthen the therapeutic window since disease onset is approximately 36 h behind that of a 7U variant challenge.

The primary objective of this experiment was to examine the difference between naïve cynomolgus macaques challenged with either 7U or 8U EBOV. Our secondary goal was to determine if there were significant differences between the two challenge groups in the context of a vaccine countermeasure, particularly a vaccine that induced immunity via EBOV GP as an antigen, since the phenotypic difference between 7U and 8U EBOV centers around ratios of sGP:GP_1,2_. Post-vaccination IgG antibody titers, the current correlate of immunity for EBOV in a number of EBOV GP-based vaccines [12,13,14,17,18,19,22], were similar to other vaccines that exhibited complete protection. Average survival differences of ≥60% between 7U and 8U EBOV were required to determine statistical significance with only eight animals per group. With protection in the 8U EBOV cohort at 87.5% while the 7U cohort was at 71.4%, these data do not support a statistical difference in efficacy amongst vaccinated cynomolgus macaques between 7U and 8U EBOV challenge. However, the decreased efficacy observed in the 7U group might be the beginning of a trend that would require a much larger population for determining statistical significance with any reliable resolution. Independent of statistical significance amongst vaccinates, it is the authors’ recommendation that future challenges be carried out utilizing a 7U EBOV in order to mitigate the risk that there is a difference between the two challenge stocks in the context of a vaccine. The reliable alternative to cell-culture propagation of EBOV challenge stocks, and the potential mutations associated with this method of propagation, would be a clonal system. A clonal system would have the added benefit of standardizing challenge material between experiments and institutes [55]; however, such an approach has its own difficulties regarding the sequence of choice for the clone. Of additional consideration is how a clone system, regardless of clone sequence choice, may potentially change viral interactions by removing biological diversity.

## Supplementary Files

Supplementary File 1
