# Supplementary material for: Ebola Virus Infections in Nonhuman Primates Are Temporally Influenced by Glycoprotein Poly-U Editing Site Populations in the Exposure Material
Source: Viruses. 2015 Dec 19;7(12):6739–54. doi: 10.3390/v7122969 (PMC4690892; doi:10.3390/v7122969)
Supplement: Supplementary File 1 [file viruses-07-02969-s001.pdf]

Supplementary Materials: Ebola Virus Infection in Nonhuman Primates is Temporally Influenced by Glycoprotein Poly-U Editing Site Populations in the Exposure Material

John C. Trefry, Suzanne E. Wollen, Farooq Nasar, Joshua D. Shamblin, Steven J. Kern, Jeremy J. Bearss, Michelle A. Jefferson, Taylor B. Chance, Jeffery R. Kugelman, Jason T. Ladner, Anna N. Honko, Dean J. Kobs, Morgan Q.S. Wending, Carol L. Sabourin, William D. Pratt, Gustavo F. Palacios and M. Louise M. Pitt

Table S1. Disease course observations for all 7U challenged NHPs. Each animal was observed cage-side for recumbence, rash, motor dysfunction and reduced appetite. The numbers in each column represent the day post-exposure (DPE) that each event was first recorded. A dash indicates that specific column's observation was not made at any point in the disease course.

Arbitrary Designation	Ebola Strain	Vaccination (✓)	Euthanasia (DPE)	Onset Primary Euthanasia Score ≥ 1 (DPE)	Rash<br>Onset (DPE)	Motor Dysfunction (DPE)	Reduced Appetite (DPE)
1	7U		7.28	5	6	6	6
2	7U		7.62	5	6	7	6
3	7U		5.65	5	5	-	5
4	7U		7.02	5	6	6	5
5	7U		5.64	5	5	-	5
6	7U		7.02	5	7	6	5
7	7U		6.36	5	6	6	5
8	7U		8.48	5	7	7	5
9	7U	✓	35.17	8	-	-	9
10	7U	✓	14.05	6	-	11	6
11	7U	✓	11.17	6	8	10	-
12	7U		6.57	5	6	-	-
13	7U	✓	7.75	6	7	-	9
14	7U		5.54	-	5	-	9
15	7U	✓	35.16	5	-	-	9
16	7U		7.53	6	6	7	-
17	7U	✓	35.66	6	-	9	-
18	7U		7.03	5	6	-	6
19	7U		5.71	5	5	-	-
20	7U	✓	35.16	-	-	-	6
21	7U		5.99	5	6	-	7
22	7U	✓	35.65	-	-	-	-

Table S2. Disease course observations for all 8U challenged NHPs. Each animal was observed cage-side for recumbence, rash, motor dysfunction and reduced appetite. The numbers in each column represent the day post-exposure (DPE) that each event was first recorded. A dash indicates that specific column's observation was not made at any point in the disease course.

Arbitrary Designation	Ebola Strain	Vaccination (✓)	Euthanasia (DPE)	Onset Primary Euthanasia Score ≥ 1 (DPE)	Rash	Motor Dysfunction (DPE)	Reduced Appetite (DPE)
					Onset (DPE)		
24	8U		6.35	5	6	6	6
25	8U		8.35	6	6	7	6
26	8U		6.47	5	6	6	4
27	8U		8.52	5	6	6	6
28	8U		8.63	6	7	7	7
29	8U		7.24	5	7	6	6
30	8U		9.02	6	7	7	6
31	8U	✓	11.01	5	10	10	9
32	8U		6.73	5	6	-	6
33	8U	✓	35.15	6	-	-	-
34	8U	✓	36.04	6	-	-	-
35	8U		9.02	5	8	9	9
36	8U	✓	36.04	6	-	-	9
37	8U		10.99	6	11	10	9
38	8U	✓	29.02	6	-	-	-
39	8U	✓	36.02	-	6	-	-
40	8U		8.35	6	6	7	6
41	8U	✓	36.03	6	-	-	-
42	8U		7.52	5	6	7	6
43	8U		8.14	6	8	8	7
44	8U	✓	36.03	6	-	-	-

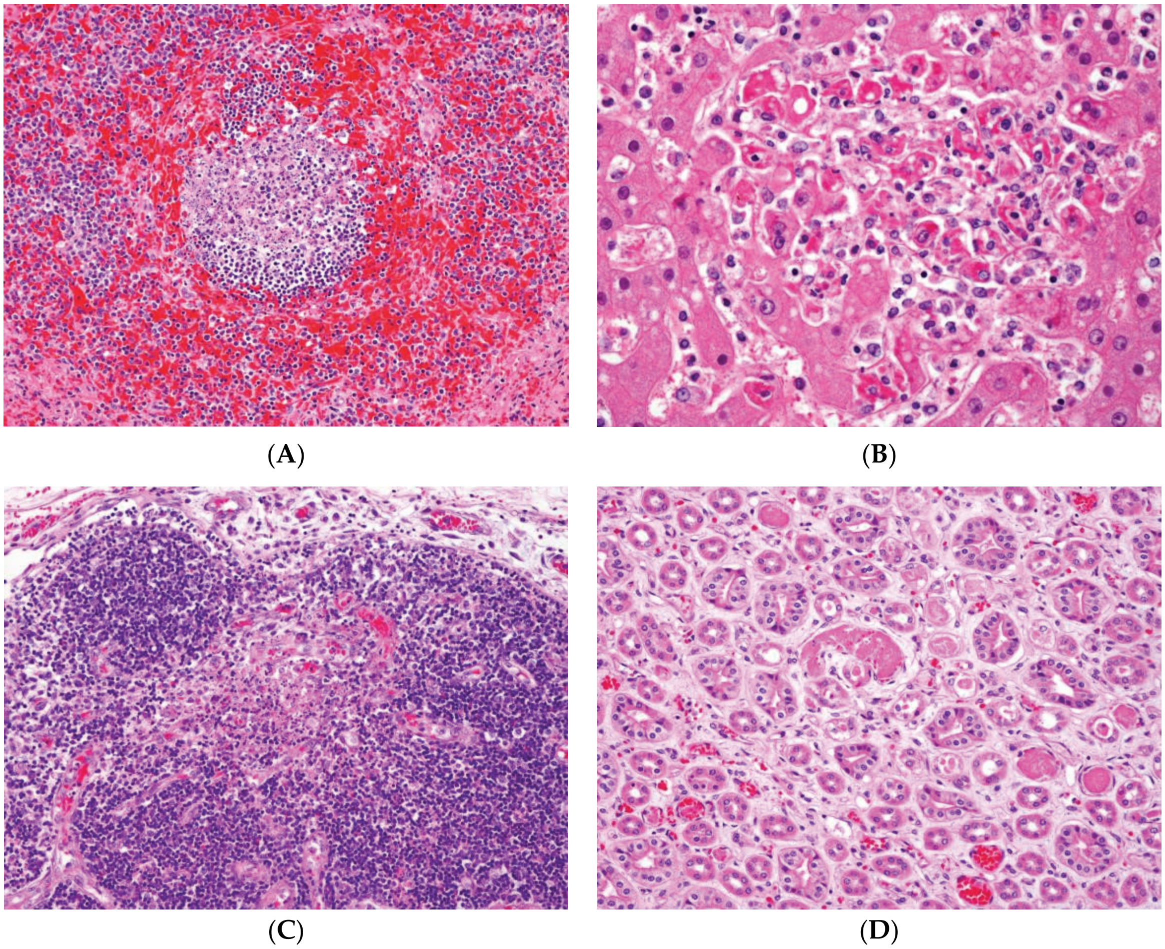

Figure S1. Pathology. All animals demonstrated similar pathological changes typical of Ebola virus disease; images (**A**)–(**D**) are examples of the pathological changes seen during hematoxyiln and eosin staining: (**A**) Spleen: Lymphocyte necrosis and lymphoid depletion; 140431, H & E, 200×; (**B**) Liver: Hepatocyte degeneration and necrosis; 140438, H & E, 400×; (**C**) Lymph node: Lymphocyte necrosis and lymphoid depletion; 140436, H & E, 200×; (**D**) Kidney: Vascular fibrin thrombi and tubular epithelial necrosis; 140435, H & E, 200×.

© 2015 by the authors; licensee MDPI, Basel, Switzerland. This article is an open access article distributed under the terms and conditions of the Creative Commons by Attribution (CC-BY) license (http://creativecommons.org/licenses/by/4.0/).
